# The Influence of Environmental Cues on the Development of *Trypanosoma cruzi* in Triatominae Vector

**DOI:** 10.3389/fcimb.2020.00027

**Published:** 2020-02-21

**Authors:** Raíssa de Fátima Pimentel Melo, Alessandra Aparecida Guarneri, Ariel Mariano Silber

**Affiliations:** ^1^Laboratório de Bioquímica de Tryps (LaBTryps), Departamento de Parasitologia, Instituto de Ciências Biomédicas, Universidade de São Paulo, São Paulo, Brazil; ^2^Vector Behaviour and Pathogen Interaction Group, Instituto René Rachou, Fundação Oswaldo Cruz, Belo Horizonte, Brazil

**Keywords:** *Trypanosoma cruzi*, Triatominae, temperature, nutritional state, oxidative imbalance, osmotic stress, host–parasite interaction

## Abstract

*Trypanosoma cruzi*, a hemoflagellate parasite, is the etiological agent of Chagas disease that affects about 6–7 million people worldwide, mostly in Latin America. The parasite life cycle is complex and alternates between an invertebrate host—Triatominae vector—and a mammalian host. The parasite adaptation to the several microenvironments through which it transits is critical to success in establishing infection. Moreover, environmental cues also play an important role on the parasite development, and it can modulate the infection. In the present study, we discussed how the temperature oscillations and the nutritional state of the invertebrate host can affect the parasite development, multiplication, and the differentiation process of epimastigote forms into metacyclic trypomastigotes, called metacyclogenesis. The impact of oxidative imbalance and osmotic stresses on the parasite–vector relationship are also discussed.

## Introduction

*Trypanosoma cruzi*, a hemoflagellate parasite belonging to the order Kinetoplastida and the family Trypanosomatidae, is the etiological agent of Chagas disease, also known as American trypanosomiasis, and this potentially lethal disease is considered one of the most neglected human diseases by the WHO (World Health Organization, 2007). Chagas disease is a key human vector-borne zoonotic disease that is endemic in 21 Latin American countries and the southern region of the United States (Bern et al., 2011; World Health Organization, 2014). In addition, new cases have been reported in Europe (Perez-Molina et al., 2011), Canada (Schipper et al., 1980), New Zealand and Australia (Jackson et al., 2014), mainly due to population mobility between the Americas and the rest of the world (reviewed in Flores-Ferrer et al., 2018). In the United States, it is also noteworthy that autochthonous human infections have been reported and that a considerable number of seropositive blood donors have been identified (Buhaya et al., 2015). Chagas disease presents an acute phase, which is mostly asymptomatic, characterized by evident parasitemia, followed by a chronic phase, characterized by the absence of evident parasitemia and a robust humoral response. Although most chronically infected people are asymptomatic, 30–40% of patients develop cardiac diseases, digestive mega syndromes, or both (Rassi et al., 2010; De Oliveira et al., 2018). The chronicity of the pathogenesis of the disease has contributed to making it difficult to diagnose, compromising treatment. In addition, discontinuities in control initiatives launched in the 1990s have been responsible for a re-emergence of Chagas disease, which became a global economic and health issue (Flores-Ferrer et al., 2018).

It is estimated that 6–7 million people worldwide are infected by *T. cruzi*, while another 65 million people, mainly in endemic areas, are at risk of acquiring the infection due to daily exposure to vector transmission (World Health Organization, 2014). In its natural cycle, the parasite is transmitted to mammals (including humans) by contaminated triatomine vectors (insects belonging to the order Hemiptera, family Reduviidae, and subfamily Triatominae). The triatomines constitute a subfamily of an otherwise predatory group of insects and comprise some 150 species. Epidemiologically, only ~20 species from the genera *Triatoma, Rhodnius*, and *Panstrongylus* are particularly relevant to *T. cruzi* transmission to humans, among which *Triatoma infestans, Triatoma dimidiata, Triatoma brasiliensis, Rhodnius prolixus*, and *Panstrongylus megistus* are considered the most important primary vectors (Gourbière et al., 2011; Guhl, 2017).

The complex life cycle of *T. cruzi* requires it to alternate between its invertebrate hosts (triatomines) and mammalian hosts. During its journey between the two kinds of hosts, the parasite encounters environments that result in physical, physicochemical biochemical, and immunological challenges. The adaptive response to such challenges results in the successful establishment of a long-lasting infection, which is critical for the transmission of the parasite to other hosts. Importantly, the responses to some of these challenges are not only related to parasite survival but can also trigger critical processes, such as differentiation to progress during the parasite life cycle; for example, stressors, such as an acidic pH or starvation may trigger the transition from one developmental stage (epimastigotes) to another (metacyclic trypomastigotes) (Jimenez, 2004). Environmental cues also play an important role in parasite development and can modulate the infection, which is supported by the existence of seasonal changes in a vector's infectivity (Asin and Catala, 1995).

In this review, we discuss how temperature oscillations and the nutritional status of the invertebrate host associated with different physicochemical properties and intrinsic factors in the microenvironment can affect parasite development and multiplication and the differentiation of epimastigote forms into metacyclic trypomastigotes (metacyclogenesis).

## Morphology and Life Cycle

**Figure 1:**
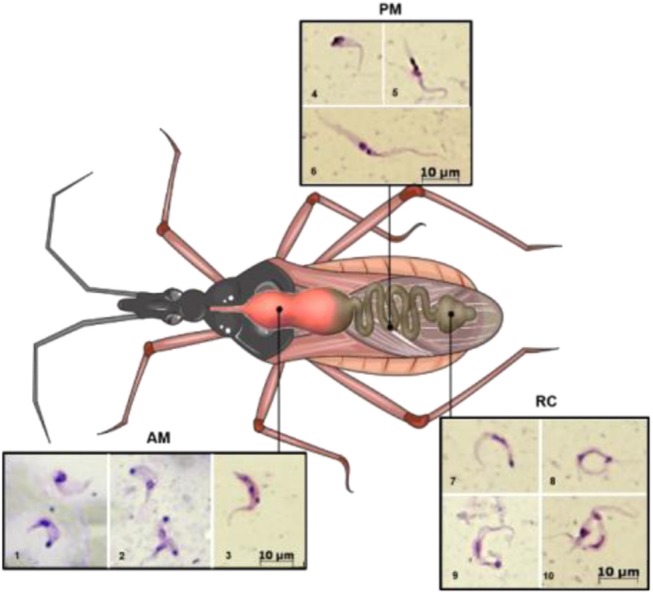
Schematic illustration showing the different developmental stages of the parasite along the digestive tract of the triatomine. AM (lower row, images 1–3), PM (upper row, images 4–6), and RC (on the right 7–10). 1, trypomastigote (below) and intermediate form; 2, amastigote-like (above) and intermediate forms; 3–5, intermediate forms; 6, epimastigote; 7–9, metacyclic trypomastigotes; 10, metacyclic and intermediate forms. AM, anterior midgut; PM, posterior midgut; RC, rectum. Photography's were obtained as follows: infected *Rhodnius prolixus* nymphs were dissected, and the parasites present in the different parts of the midgut were collected, Giemsa stained, and photographed under light microscopy. This figure was created using a triatomine image from Servier Medical Art Commons Attribution 3.0 Unported License (http://smart.servier.com). Servier Medical Art by Servier is licensed under a Creative Commons Attribution 3.0 Unported License.

During its life cycle, *T. cruzi* undergoes changes in its morphology as well as its biochemical and biological properties (such as infectivity and the ability to proliferate). In the intestinal tract of the kissing bug, three main stages are found (Chagas, 1909)—epimastigotes, trypomastigotes, and spheromastigotes—as well as many intermediate stages, which can be generically described as flagellates with either a drop-like shape (intermediate between spheromastigotes and epimastigotes or trypomastigotes) or a slender shape (intermediate between epimastigotes trypomastigotes) (Schaub, 1989), as represented in Figure 1. Epimastigotes are able to multiply and colonize the intestinal tract of the vector. Metacyclic trypomastigotes (non-replicative forms) develop in the rectum and are infectious in mammals (Kollien and Schaub, 1999). In the mammalian host, intracellularly multiplying amastigotes are present, and as a result of successive binary fissions, they develop into non-replicative trypomastigotes (Tyler and Engman, 2001) via an intermediate transient epimastigote-like stage (also referred to in the literature as intracellular epimastigotes) (Almeida-de-Faria et al., 1999; Tyler and Engman, 2001).

**Figure 2:**
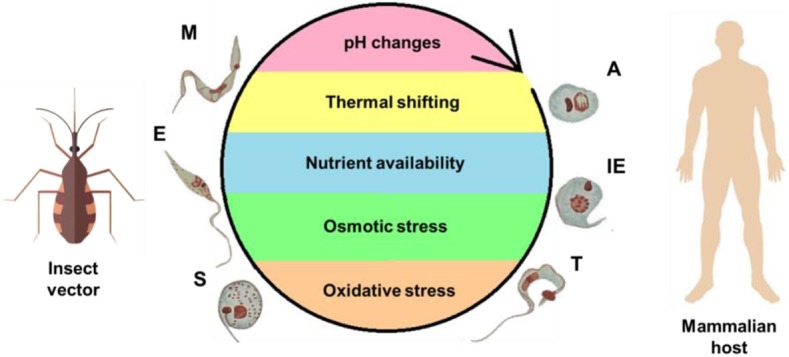
Life cycle of *T. cruzi* and the stress factors encountered during its development. To complete its complex life cycle, the parasite alternates between an invertebrate host—the insect vector—and mammalian host. Different microenvironments are encountered, resulting in stress to the parasite, as represented within the cycle. The forms are abbreviated as follows: A, amastigote; E, epimastigote; IE, intracellular epimastigote; M, metacyclic trypomastigote; S, spheromastigote; T, trypomastigote.

During a bloodmeal, the infected triatomine insect sucks a significant amount of blood from the mammalian host, forcing the elimination of excrement from the insect's rectum and releasing metacyclic trypomastigotes. The parasites then contact the injured skin or mucosa and are internalized into this new host. As trypomastigote forms are not replicative, the establishment of the infection depends on the ability of the parasite to differentiate into a replicative stage, which only occurs inside mammalian host cells. For this reason, they must invade host cells and reach the cytosol, where they differentiate into amastigotes (De Souza et al., 2010). After several cycles of cell division, amastigotes differentiate into intracellular epimastigotes (Almeida-de-Faria et al., 1999) and then into trypomastigotes, which burst from infected cells into the extracellular environment. These parasites can infect neighboring cells or reach the bloodstream, where they can spread the infection to other tissues, or they can be ingested by a kissing bug during its bloodmeal, and the bug will then transmit the infection to other hosts (reviewed in Marchese et al., 2018). The classic description of the parasite's development in the digestive tract of the insect (Chagas, 1909; Dias, 1934), in which blood trypomastigotes differentiate into epimastigotes in the stomach and then multiply continuously in the intestine, reaching the rectum, where they differentiate into metacyclic trypomastigotes, was initially refuted by Brack. The author suggested that bloodstream trypomastigotes differentiate into rounded forms with free flagella referred to as spheromastigotes, which then multiply and differentiate into trypomastigotes or epimastigotes (Brack, 1968). In fact, Brener observed a large number of rounded or slightly pear-shaped parasites in stomach content samples ~72 and 96 h after infection. It has also been suggested that a different process of reproduction could occur in the anterior midgut (AM) with some degree of genetic exchange, characterized by the fusion of amastigote forms, followed by the apparent reorganization of DNA-containing organelles and subsequent detachment of new flagellates (Brener, 1972). The process in which the bloodstream trypomastigotes differentiate into epimastigotes is referred to as epimastigogenesis and apparently occurs in the posterior midgut (PM), where they initiate their replication. It is important to note that, although there is a paradigm concerning this issue, it has recently been shown that both trypomastigote stages of *T. cruzi* (cell-derived and metacyclic forms) are able to transform into epimastigotes. Interestingly, these “recently differentiated epimastigotes” exhibit relevant biological properties, such as resistance to complement-mediated lysis and both *in vitro* (cell culture) and *in vivo* (mouse) infectivity. This indicates dynamic behavior in which both metacyclogenesis and secondary epimastigogenesis can occur in the triatomine rectum (Kessler et al., 2017). Midgut colonization by *T. cruzi* was recently revisited by Ferreira and collaborators, and as reported by Dias (1934), epimastigogenesis was shown to be completed in the PM of *R. prolixus* (Ferreira et al., 2016) and not in the anterior midgut, as was assumed for quite some time. Furthermore, the AM seems to be an inhospitable environment for the parasite, since the trypomastigote population is severely reduced 24 h after invading this portion of the gut (Dias et al., 2015; Ferreira et al., 2016; reviewed in Guarneri and Lorenzo, 2017). During this initial interval, it is assumed that the remaining parasites differentiate into intermediate or amastigote-like forms and quickly travel to the PM, where they will start to replicate (Guarneri and Lorenzo, 2017). At later stages in the rectum, some proportion of epimastigotes attach to the rectal cuticle as a prerequisite step to initiate metacyclogenesis (Garcia and Azambuja, 1991; Kollien and Schaub, 2000; Azambuja et al., 2005; Garcia et al., 2010). Briefly, the life cycle of *T. cruzi* (with the main stages) can be schematically illustrated as follows (Figure 2).

## Environmental Cues and Their Impact on Parasite Development in the Insect Host

The multiple environmental changes encountered by *T. cruzi* are a consequence of the different organisms (and multiple tissues therein) in which its life cycle takes place. This fact is probably closely related to the ability of *T. cruzi* to sense and adapt to these environments, maintain their cell homeostasis, and, ultimately, survive to extremely different physical, physicochemical, chemical, and nutritional conditions (Zuzarte-Luís and Mota, 2018). The main environmental variables that *T. cruzi* encounters during its life cycle are represented schematically (Figure 2) and include variations in temperature, fluctuations in the type and availability of nutrients, and changes in pH, osmolarity, ionic composition, and redox potential (Jimenez, 2004).

The influence of the insect vector gut microbiota on the parasite life cycle is an important issue. It is well-established that the composition of the intestinal microbiota can interfere with the effectiveness of the infection. Importantly, an inadequate balance between the bacterial and protozoan populations can compromise the establishment of the infection, since the two populations compete for resources in the intestine (De Oliveira et al., 2018). Previous studies by Azambuja and collaborators have shown that a few days after blood feeding, the number of bacteria in the insect AM from *R. prolixus* increases considerably, leading to the lysis of erythrocytes and the *T. cruzi* Y strain (Azambuja et al., 2004, 2005). A recent posterior study demonstrated that the incubation of the *T. cruzi* Y strain with the bacterium *Serratia marcescens* led to parasite lysis. This effect was dependent on the ability of the bacterial strain to adhere to the protozoan surface via D-mannose-recognizing fimbriae. This seems to be a strain-dependent phenomenon, since different bacterial strains show different behaviors. The bacterium–protozoan attachment seems to result in a filamentous biofilm, which is critical for parasite–microbiota interactions in the gut of triatominae (Castro et al., 2007).

Keeping in mind the general context of this work, it is crucial to understand that environmental conditions have been recognized as being key to the dynamics of infectious diseases, affecting parasite transmission and virulence (Wolinska and King, 2009). In the case of *T. cruzi*, which colonizes different parts of the triatomine digestive tract, the environment varies in space and time, and many peculiarities of this variation can shape the outcome of insect infection. Changes in temperature can modulate the spread of the infection and exert profound and complex ecological feedback on its dynamics, for example, by changing the availability and quality of nutritional resources for the host (Cornet et al., 2014).

### Temperature Variation

Similar to all terrestrial organisms, triatomines inhabit thermally heterogeneous environments, and this heterogeneity is a consequence of spatial (at large or small scales) and temporal variations (over years, months, seasonally, or during a single day). It is well-established that changes in temperature affect biological processes. The impact of such temperature changes on ectothermic animals, such as insects is greater because of their inability to control their body temperature (Rolandi and Schilman, 2018).

A recent study from Fellet et al. showed that the fecundity and fertility rates of *R. prolixus* are affected by the presence of *T. cruzi* depending on the temperature at which insects are maintained. The authors developed an experiment in which insects infected by *T. cruzi* in the second-instar nymph stage were maintained in a chamber at 25°C during nymphal development and after imaginal molting (this is a reference to differentiation to the adult phase, also referred to as the imaginal stage), or were maintained in a chamber at 30°C during nymphal development and transferred to 25°C after imaginal molting. In the first group, the capacity of the females to convert ingested blood into eggs (*e* value) was increased by the infection depending on the adult age, as was the hatching rate. For the second group, the analysis of the *e* values showed that the production of eggs was decreased in infected insects during the first reproductive cycle. However, the fertility of the eggs was decreased only in the third reproductive cycle (Fellet et al., 2014). In another study, *T. cruzi*-infected *R. prolixus* were maintained at four different temperatures (20, 24, 27, or 30°C), and a considerable delay in the time at which the insects triggered the molting process was observed across the temperatures. This extension of the intermolt period would be beneficial to the parasites, as they need time to develop inside the insect before the next bloodmeal (in nymphs that feed to completion, the next bloodmeal only occurs after the molt). Parasite infection was also found to increase mortality rates at two intermediate temperatures, 24 and 27°C, but not at 21 or 30°C. It is important to note that, in this study, the insects were observed for 90 days, during which they did not receive additional bloodmeals. The lack of effects observed in the insects kept at 21°C was possibly related to a slowdown in insect metabolism imposed by the temperature, which maintained the nutritional resources at sufficient levels to allow the development of the parasite without affecting insect survival. At the other extreme, the highest temperature increased insect metabolism, leading to the rapid exhaustion of the energetic resources, which probably eliminated the parasites (Elliot et al., 2015).

In triatomine bugs, the prophenoloxidase (proPO) and phenoloxidase (PO) enzymatic cascade plays a role in the immune response related to defense against pathogens. In this regard, *Meccus pallidipennis*, a vector with high epidemiological importance in Mexico, was used to determine the activity of the proPO system against two *T. cruzi* strains in different intestinal regions and temperature conditions. In general, with increasing temperature (20–34°C), proPO activity decreased. In contrast, for the PO system, the highest activity values were observed at 30°C, and they were lower at 34°C. The more efficient proPO activity before a critical increase in temperature could be explained by the fact that this system works better at moderately higher temperatures but not when the temperature reaches a lethal limit. Increased temperatures negatively affect the triatomine life cycle. The presence of *T. cruzi* decreases insect survival, and this phenomenon is related to the strain and temperature. The insects are more affected by the Chilpancingo strain than by the Morelos strain, and these effects are even more perceivable at higher temperatures (34°C), suggesting a synergistic effect between temperature and the parasitic strain on insect survival (González-Rete et al., 2019).

In addition to changes in the physiology of triatomines, early studies have suggested that temperature has significant effects on the parasite–vector interaction, influencing the development of *T. cruzi* within the insect. According to Neves, who qualitatively characterized the parasite's life cycle in *T. infestans* at temperatures ranging from −5 to 37°C, complete parasite development occurs between 23 and 28°C (Neves, 1971). In addition to its direct influence, temperature may modulate parasite development by modifying some physiological processes of the vector, which is linked to variations in the *T. infestans* blood consumption (Catala et al., 1992) and could modify the environment in which the parasite multiplies and differentiates (Asin and Catala, 1995). However, it should be taken into account that these experiments refer to a description based on non-infected insect physiology.

The optimum temperature for the development of most triatomine species is 24–28°C (Jurberg and Galvão, 2006). Previous studies published by Wood demonstrated an increase in the parasite number and the presence of more active metacyclic and non-metacyclic forms when the excreted content of the insect vector was analyzed during warmer periods of the year compared to colder ones (Wood, 1938, 1941, 1943; Woon, 1942). Subsequently, a study by the same author reported that lower environmental temperatures (22–23°C) decreased the release of metacyclic forms of *T. cruzi* from newly infected adult *T. protacta*, while higher environmental temperatures (28–34.5°C) increased their release. Under a higher temperature, metacyclic trypomastigotes appeared in vector feces on the 7th day after the infective blood meal, but they were not found under the lower temperature, at least until the 12th day. At this temperature, the fecal samples examined 36 days after the infective meal revealed few metacyclic forms (Wood, 1954). Finally, Phillips noted that in *R. prolixus*, metacyclic trypomastigotes can be found as early as the 2nd day after feeding on infected blood when the experimental temperature is 30°C, whereas they appear on the 7th day after the infective meal when the insects are maintained at 20°C (Phillips, 1960). Considering the different experimental conditions in which these studies were performed (regarding triatomine species, parasite strains, and the analyzed time period, among other factors), it is possible to conclude that the temperature dependence shows a similar pattern in all cases: lower temperatures delay the release of metacyclic trypomastigotes in feces, while at higher temperatures, infectious forms develop earlier.

Importantly, it is well-established that temperature affects the proliferation of epimastigotes. However, a small number of studies have provided evidence of how important this factor is for parasite proliferation in the triatomine host. Asin and Catalá conducted this evaluation by maintaining infected kissing bugs at 20 and 28°C. In this study, *T. infestans* had daily opportunities to consume blood, which is not the usual situation in nature. Although epimastigotes started to proliferate immediately at both temperatures, the epimastigote density increased slowly at 20°C compared to 28°C. Metacyclogenesis was also affected by the temperature change, since the presence of metacyclic forms within the insects' recta and feces was delayed at 20°C. Once differentiation into the infective stage was completed, the density of the metacyclic trypomastigotes in feces was similar at the two temperatures. As observed in the above-mentioned studies, this work also showed that a suboptimal temperature retards *T. cruzi* differentiation within the vector (Asin and Catala, 1995).

Data regarding the proliferation of epimastigotes *in vitro* have been reported recently, indicating that epimastigotes grow optimally at a temperature of 28°C. The temperatures of 33 and 37°C were also evaluated, and it was observed that cells grew to a higher density at 33°C, while cell growth at 37°C was slightly greater than that observed at 28°C when CL strain clone 14 was used (Magdaleno et al., 2009), indicating that the optimal temperature may be strain dependent. In another study involving the CL strain, the epimastigote proliferation rate was found to increase together with the environmental temperature, with peak growth occurring at or above 30°C. Increased proliferation rates could have evolved as a “strategy” for increasing the parasite population, thus increasing the chances of transmission (due to a high population density in the intestine), even with the possible cost of killing the vector and interrupting transmission (Elliot et al., 2015).

### Feeding Habits of Triatomines and Their Relationship With Oxidative Imbalance and Osmotic Stress

Since *T. cruzi* lives in the intestinal tract of a triatomine, the parasite may be affected by changes in the nutritional supply, i.e., by the ingestion of blood or starvation (Kollien and Schaub, 1999). In this context, some variables associated with the biology of the insect vector are important, such as the periodicity of feeding and the amount of blood that is taken in each bloodmeal. *In natura*, triatomine insects are able to go for months without feeding. In these cases, when they have the chance of taking a bloodmeal, they usually ingest a relatively large amount of blood; *R. prolixus* nymphs, for example, take approximately nine times their body weight in blood (Friend et al., 1965). A very interesting insight that may explain this behavior is found in a paper published by Sterkel et al., in which the authors compare the average weight of a mosquito and a human, noting a size difference of ~4 × 10^7^. The authors explain that “the defensive behavior of vertebrates provides a major protective effect against blood-sucking insects and makes feeding extremely dangerous for most hematophagous invertebrates. Consequently, the minimization of the number of visits to the vertebrate host is a common trend in the biology of most of these animals and is accomplished in large part by the ingestion of disproportionally large amounts of blood in a single meal” (Sterkel et al., 2017).

In triatomines, the body shape changes and the abdomen dilates considerably as soon as the insects begin their meal, since cuticle plasticization leads to the extension of the abdominal integument, allowing the accommodation of large amounts of blood in the anterior midgut (Bennet-Clark, 1962). Blood ingestion induces rapid changes in the rectum in particular. The large amount of blood ingested creates a number of challenges for the maintenance of homeostasis, impacting triatomine biology, increasing the weight of the insects, and affecting their movements. To cope with these challenges, shortly after the bloodmeal, triatomines reduce their weight by releasing excreta and large amounts of water. This is possible due to their extraordinarily efficient excretory system, which allows most of those compounds taken in with the blood that have little or no nutritional value (i.e., water and ions, including large quantities of Na^+^ and Cl^−^) to be expelled in a short time. Nutrient-rich blood cells are simultaneously concentrated (Maddrell, 1972). This phenomenon can be observed in *R. prolixus*, in which the secretory rate of the Malpighian tubules is increased by a factor of ~1,000 times after a bloodmeal (Maddrell, 1969). This rapid nutritional transition that occurs in the intestinal tract of the insect vector impacts the osmolarity of the surrounding environment and consequently results in osmotic stress to the parasite. One study demonstrated that after feeding, the first clear urine that was produced was generally almost isotonic with respect to the ingested blood and somewhat hypo-osmotic with respect to the hemolymph. Approximately 2.5 h later, the osmolarity of the urine increased slightly, and at 24 and 48 h after the bloodmeal, the osmolality of the urine had doubled, which represents a very hostile change for the parasite (Wigglesworth, 1931; Kollien et al., 2001). This pattern may be related to the fact that, in the long term, the digestion of blood cells leads to an excess of ions, such as K^+^ and Ca^2+^ as well as nitrogen-containing waste metabolites (uric acid and organic anions) resulting from the catabolism of bloodmeal proteins. In addition, the toxins present in the diet or produced via blood metabolism must be excreted (reviewed in O'Donnell, 2009).

The chemical composition of the large amount of vertebrate blood ingested by triatomine insects includes proteins, which account for almost 90% of the dry weight of vertebrate blood, among which hemoglobin is the most abundant (~150 mg/ml) (Sterkel et al., 2017). The contents of the blood are continuously digested via the action of proteases, releasing amino acids, small peptides, and heme. Potential hyperamino acid toxicity is prevented by protein digestion coupled to fast oxidative degradation pathways that convert amino acids into molecules that contribute to gut homeostasis. Heme is released mostly through the breakdown of hemoglobin. Similar to other trypanosomatids, *T. cruzi* is not able to synthesize heme because the complete biosynthesis pathway is absent. Taking into account the essentiality of heme, parasites are strictly dependent on its uptake from their hosts (Cupello et al., 2011; Merli et al., 2016), after which it is inserted into different heme proteins (reviewed in Merli et al., 2017). The high heme concentrations resulting from blood digestion have shaped some aspects of the gut cell biology of triatomines, such as the control of intracellular heme levels by microsomal heme oxygenase (HO). In *R. prolixus*, a unique heme-degradation pathway has been described. Heme is first modified by the addition of two cysteinyl glycine residues, and the porphyrin ring is then cleaved. Finally, the dipeptides are trimmed, resulting in the production of dicysteinyl-BV IX, CO, and iron (Paiva-Silva et al., 2006). A reduced but still significant amount of heme reaches the cytosol of midgut cells and the hemolymph, putting tissues at risk of oxidative damage because this can lead to the generation of reactive oxygen species (ROS) through the Fenton reaction. The Fenton reaction consists of the conversion of H_2_O_2_ into hydroxyl radicals, which are among the most potent known oxidants, using electrons donated by the Fe^2+^ atom present in the heme nucleus. These ROS can potentially damage biological molecules: they can inactivate proteins, disrupt the phospholipid bilayer of cell membranes, and damage DNA, which results in toxicity to the parasites (Schmitt et al., 1993). However, it is worth reinforcing that *T. cruzi* is strictly dependent on heme as a nutritional cofactor for its metabolism and as an integral component of a series of essential heme proteins (Lara et al., 2007). The need for a “dangerous” metabolite, such as heme that can accumulate in large quantities in the same niche inhabited by the parasite led us to think about possible fine-tuning strategies involved in the interaction between the parasite and its insect host. Parasites belonging to the genus *Plasmodium*, which are the etiological agents of malaria, are able to digest host-cell hemoglobin. They detoxify the excess free heme in the parasite's food vacuole by polymerizing it into a harmless dark-brown crystalline structure referred to as malaria pigment or hemozoin (Hz) (Slater et al., 1991). More recently, Oliveira et al. demonstrated that this also occurs in the midgut of the bloodsucking insect *R. prolixus*. The authors showed by transmission electron microscopy (TEM) that large electron-dense aggregates that were similar in appearance to the Hz granules found in *Plasmodium* parasites existed in the lumen of the *R. prolixus* midgut. Finally, their chemical nature was determined, demonstrating the hypothesis of heme detoxification through its conversion to hemozoin in triatomines. It was shown that through this mechanism, these insects can detoxify more than 97% of the heme that is present and that this process occurs in the perimicrovillar membranes, which are extracellular lipid membranes that separate the gut epithelium from the luminal content in hemipterans. This finding is very important, and this process can be considered the first line of defense against the effects of the release of heme via hemoglobin digestion. The sequestration of heme in an insoluble form leads to its elimination in the insect's feces, preventing the heme from crossing the midgut wall and causing oxidative tissue damage (Oliveira et al., 1999; Sterkel et al., 2017). Interestingly, Hz synthesis in the midgut of insects is promoted by a particulate fraction from the intestinal lumen, and the factor responsible for its synthesis is heat labile. To better understand this mechanism, *R. prolixus* were fed with blood supplemented with different concentrations of chloroquine (CLQ) as well as known potent inhibitors of Hz formation. As a result, the heme concentration in the hemolymph increased, which resulted in higher lipid peroxidation. This report reinforces the importance of this mechanism involved in the crystallization of Hz in the *R. prolixus* midgut as a physiological defense against heme toxicity (Oliveira et al., 2000).

### Nutritional Availability and Its Impact on Parasite Development

As previously reported, in their natural environment, triatomines go for weeks between feedings, and the complete digestion of a blood meal can take up to 10 days. The influence of starvation on the *T. cruzi*–triatomine interaction has been analyzed by different authors, and a reduction in the number of parasites and the appearance of dead flagellates in starved insects have been reported. In fact, the lack of nutrients affects the population density of *T. cruzi* and, depending on the time interval, results in parasite death. The first dead flagellates can be detected in the rectum after short-term starvation (30 days), while long-term starvation (90 days) can kill up to 99.5% of the *T. cruzi* population in the rectum (Kollien and Schaub, 1998, 2000). Moreover, starvation affects different developmental stages of *T. cruzi* as well as the course of metacyclogenesis differentially. Fifth-instar *R. prolixus* larvae subjected to fasting for different times have been shown to exhibit different rates of differentiation. The lowest percentage of differentiation occurred in a group subjected to 45 days of starvation. This group presented 45% of the normal level of metacyclogenesis, while the others (15 and 30 days of starvation) presented differentiation rates of 85 and 65%, respectively (Garcia et al., 1995).

Kollien and Schaub also demonstrated that when *T. infestans* was parasitized by *T. cruzi*, after a starvation period of 20 or 30 days, the population of flagellates decreased substantially, and at 60 days after the last feeding (daf), no flagellates were found in the small intestine. The total rectal population was reduced by one-third between 30 and 60 daf, followed by a more significant reduction over the next 30 days, and changes also occurred in different stages, while in regularly fed triatomines, the parasite population in the rectum consisted mainly of equal amounts of epi- and trypomastigotes. The percentage of trypomastigotes seemed to be unaffected by the insect starvation period, and a similar trend was observed on the rectal wall. On the other hand, the percentage of epimastigotes in the rectal lumen and on the rectal wall decreased at 20 daf (from 50 to 15–30%). At ~90 daf, this rate reached 50% on the rectal wall. Interestingly, the percentages of slender intermediate forms varied little during different starvation periods, while drop-like intermediate forms showed a continuous significant increase during the starvation process (ranging from 1 to ~15%). The percentages of spheromastigotes also increased at 60 daf, reaching 22% in the lumen and 18% on the wall. It is important to point that in recently fed bugs that were previously subjected to starvation, spheromastigotes (and other intermediate forms) almost disappear, while epimastigotes dominate (Kollien and Schaub, 1999). This pattern may be associated with the role of spheromastigotes as a stage in the parasite life cycle that develops under stress conditions (Schaub, 1989; Kollien and Schaub, 1998). Feeding the vector at 40 daf induced the appearance of pure populations of trypomastigotes in immediately deposited drops of bug urine and induced metacyclogenesis in epimastigotes. These data emphasize the importance of the feeding status of the vector for the development of different stages of *T. cruzi*.

Interestingly, it has been proposed that blood consumption could regulate epimastigote population density and thereby influence epimastigote differentiation to metacyclic forms, or metacyclogenesis. A study published by Asin and Catalá showed that in *T. infestans*, blood consumption is related to the development of *T. cruzi* epimastigote and metacyclic trypomastigote forms at 28°C. The consumption of at least 120–180 mg of fresh blood ensures the development of the parasite, while a lower amount of consumption impacts this process. However, it does not influence the number of trypomastigotes released in the feces (Asin and Catala, 1995). Asin also observed that nymphs of *T. infestans* that were fed only once did not possess rectal trypomastigotes and that the total parasitic population declined (Asin, 1992). As mentioned above, triatomines usually consume a large amount of blood in a single feeding, resulting in high hemoglobin ingestion. Garcia et al. demonstrated that when plasma was supplemented with high hemoglobin concentrations and offered to fifth-instar *R. prolixus* nymphs as a meal, there was an increase in the *T. cruzi* metacyclogenesis rate compared to that in insects fed only whole blood or plasma. Therefore, hemoglobin not only has a nutritional effect but is also important in the induction of differentiation (Garcia et al., 1995).

In summary, different natural stresses, such as those caused by temperature changes, the availability of nutrients, or environmental changes in the redox state of *T. cruzi* habitats inside the insect vector critically influence parasite biology. Myriad adaptations have been selected over time due to the long-term interaction driving coevolution. The consequent exceptional metabolic flexibility of *T. cruzi* is an evolutionary response to the constant challenges it faces in both hosts, but particularly in the insect midgut, which provides a non-homeostatic environment.

## Concluding Remarks

Although several studies have explored the relationship between environmental cues and their impact on the development and biology of a variety of triatomine species (affecting characteristics, such as fertility, mortality, and survival), the way in which parasites respond to these environmental changes has not been explored in depth, especially through *in vitro* experiments, providing little support for discussions of this subject. The purpose of this paper was precisely to collect the main reports in the literature that describe how external factors, such as temperature, the availability of nutritional contents, and consequent oxidative and osmotic stresses may alter the development of *T. cruzi* forms in the insect vector. It has been shown that these stress factors can modify most of the critical processes required for the establishment and transmission of the infection, including the replication of epimastigote forms, the process of metacyclogenesis, the abundance of metacyclic forms and their release in insect feces, the parasitic load, and the parasite composition (percent of the different life cycle stages) in the triatomine gut. It is foreseeable that some issues related to the topics detailed in this review will garner more attention in the literature. The effect of temperature changes on *T. cruzi* development in its vector and transmission to mammals may be important for analyzing possible changes in transmission patterns due to climate change (Paaijmans et al., 2010). Some other types of complex ecological feedback could also be interpreted in this light, such as the possible alterations of the availability and quality of nutritional resources consumed by the insect host (Cahill et al., 2013). Changes in host nutritional status lead to repercussions regarding the dynamics of infection, as they may limit or modify the parasite's access to the nutritional content of the host, upon which it depends to complete its life cycle.

## Author Contributions

RM and AS conceived the manuscript. RM wrote the first version of the manuscript, produced the figures, and corrected the manuscript. AG and AS worked on the majority of the corrections of the manuscript and the figures. AS prepared the final version of the manuscript.

### Conflict of Interest

The authors declare that the research was conducted in the absence of any commercial or financial relationships that could be construed as a potential conflict of interest.
